# Extracorporeal Cardiac Shock Wave-Induced Exosome Derived From Endothelial Colony-Forming Cells Carrying miR-140-3p Alleviate Cardiomyocyte Hypoxia/Reoxygenation Injury *via* the PTEN/PI3K/AKT Pathway

**DOI:** 10.3389/fcell.2021.779936

**Published:** 2022-01-10

**Authors:** Dan Yang, Mingqiang Wang, Zhao Hu, Yiming Ma, Yunke Shi, Xingyu Cao, Tao Guo, Hongbo Cai, Hongyan Cai

**Affiliations:** ^1^ Department of Cardiology, The First Affiliated Hospital of Kunming Medical University, Kunming, China; ^2^ Department of Cardiology, Yunnan Fuwai Cardiovascular Hospital, Kunming, China; ^3^ Department of Vascular Surgery, The First Affiliated Hospital of Kunming Medical University, Kunming, China

**Keywords:** extracorporeal cardiac shock waves, cardiomyocyte hypoxia/reoxygenation injury, endothelial colony-forming cells, exosomes, miR-140-3p

## Abstract

**Background:** Stem cell-derived exosomes have great potential in the treatment of myocardial ischemia–reperfusion injury (IRI). Extracorporeal cardiac shock waves (ECSW) as effective therapy, in part, could activate the function of exosomes. In this study, we explored the effect of ECSW-induced exosome derived from endothelial colony-forming cells on cardiomyocyte hypoxia/reoxygenation (H/R) injury and its underlying mechanisms.

**Methods:** The exosomes were extracted and purified from the supernatant of endothelial colony-forming cells (ECFCs-exo). ECFCs-exo treated with shock wave (SW-exo) or without shock wave (CON-exo) were performed with high-throughput sequencing of the miRNA. H9c2 cells were incubated with SW-exo or CON-exo after H/R injury. The cell viability, cell apoptosis, oxidative stress level, and inflammatory factor were assessed. qRT-PCR was used to detect the expression levels of miRNA and mRNA in cells and exosomes. The PTEN/PI3K/AKT pathway-related proteins were detected by Western blotting, respectively.

**Results:** Exosomes secreted by ECFCs could be taken up by H9c2 cells. Administration of SW-exo to H9c2 cells after H/R injury could significantly improve cell viability, inhibit cell apoptosis, and downregulate oxidative stress level (*p *< 0.01), with an increase in Bcl-2 protein and a decrease in Bax, cleaved caspase-3, and NF-κB protein (*p *< 0.05). Notably, miR-140-3p was found to be highly enriched both in ECFCs and ECFCs-exo treated with ECSW (*p *< 0.05) and served as a critical mediator. SW-exo increased miR-140-3p expression but decreased PTEN expression in H9c2 cells with enhanced phosphorylation of the PI3K/AKT signaling pathway. These cardioprotective effects of SW-exo on H/R injury were blunted by the miR-140-3p inhibitor. Dual-luciferase assay verified that miR-140-3p could directly target the 3′UTR of PTEN mRNA and exert a negative regulatory effect.

**Conclusion:** This study has shown the potential of ECSW as an effective stimulation for the exosomes derived from ECFCs *in vitro*. SW-exo exerted a stronger therapeutic effect on H/R injury in H9c2 cells possibly *via* delivering exosomal miR-140-3p, which might be a novel promising strategy for the myocardial IRI.

## Introduction

Acute myocardial infarction (AMI) is a serious killer threatening human health and life with a high rate of morbidity and mortality worldwide (Reed et al., 2017). Although timely intervention including thrombolytic therapy, primary percutaneous coronary intervention (PPCI), coronary artery bypass grafting (CABG), and other emergency measures are taken to rescue infarcted myocardium, the process of reperfusion could induce further damage known as myocardial ischemia–reperfusion injury (IRI) (Eltzschig and Eckle, 2011; Bulluck et al., 2016). Hypoxia/reoxygenation (H/R) injury results in a series of pathophysiological changes within the myocardium, leading to myocardial stunning, energy metabolism disorder, and microvascular dysfunction (Hausenloy and Yellon, 2013). Clinical studies suggested that patients with myocardial IRI were closely related to poor prognosis owing to their aggravated progression of heart failure and worsening ventricular remodeling (Stone et al., 2016). In this regard, some promising approaches to multitargeted cardioprotective therapy were proposed in order to reduce myocardial IRI (Davidson et al., 2019). The combination drug therapy, cell therapy, ischemic conditioning, and physical hypothermia therapies were applied on clinical trials, but the therapeutic effect is not ideal in some cases (Hausenloy et al., 2017).

In recent years, stem cell therapy has been considered one of attractive therapeutic approaches for the treatment of a variety of diseases particularly in cardiovascular diseases (CVDs) (Goradel et al., 2018). Many studies have shown that stem cells have the repair and regeneration effects on CVDs *via* vein or myocardium injection including mesenchymal stem cells (MSCs) (Bartolucci et al., 2017), embryonic stem cells (ESCs) (Menasché et al., 2015), endothelial progenitor cells (EPCs) (Ward et al., 2007), and induced pluripotent stem cells (iPSCs) (Musunuru et al., 2018). However, stem cell therapy in the clinic has been limited by poor survival rates of transplanted cells, potential immunogenicity, and tumorigenesis, as well as concerns about the difficulties of cell storage and transportation (Der Sarkissian et al., 2017). Surprisingly, stem cell-derived exosomes were observed to have more advantages than cell therapy, which can replicate many of the same cardioprotective effects of stem cell and allow the transfer of biological contents locally and distally (Sahoo et al., 2011; Arslan et al., 2013). Moreover, the cargo of exosomes can be modified by pretreatment or artificial manufacture to make it more efficient for targeted therapy (Kooijmans et al., 2012; Feng et al., 2014), but exosome therapy also has several limitations, such as low and unstable amount, cumbersome extracted processes, and unclear mechanism.

Currently, there has been growing interest in activating exosomes derived from many types of cells by different stimuli. The secretion of exosomes or exosomal cargoes may be affected by internal environment and external stimuli. It has been shown that shock wave therapy can induce the release of exosomes resulting in the improvement of myocardial fibrosis and left ventricular function in the post-ischemic heart (Gollmann-Tepeköylü et al., 2020). Vicencio found that the exosomes extracted from the plasma of remote ischemic preconditioning (RIPC) can reduce the infarct size in rats with myocardial IRI (Vicencio et al., 2015). Besides, exosomes secreted by cardiomyocytes subjected to ischemia have also been proven to promote cardiac angiogenesis (Ribeiro-Rodrigues et al., 2017). Long-term exercise exosomes derived from EPCs have stronger restorative effects on H/R damage of endothelial cells (Ma et al., 2018) or mouse ischemic stroke (Wang et al., 2020b). For this reason, stimulation of exosome-producing cells might be a practicable alternative to exert myocardial protective effects.

Extracorporeal cardiac shock waves (ECSW) is a safe, effective, noninvasive, and promising new therapy for the treatment of cardiovascular diseases (Kikuchi et al., 2018; Ceccon et al., 2019). Our previous study confirmed that the cardioprotective effects of ECSW therapy have been achieved mostly by promoting endothelial progenitor cell proliferation, mobilization, and activation (Cai et al., 2015). Endothelial colony-forming cells (ECFCs), an endothelial lineage of EPCs, can be considered true EPCs with high proliferative potential and angiogenic properties (Medina et al., 2017). As the key effector cells of ECSW, the ECFCs are gradually being recognized for their benefits that are usually attributed to the paracrine activity of the cells by secreting functional exosomes (Burger et al., 2015). Most miRNAs were found in exosomes, indicating that the exosome might be the carrier of miRNAs to mediate information transmission between cells. ECSW may induce ECFCs to release activated exosomes playing regulatory roles in heart repair, but it still remains poorly understood. Therefore, this study set out to explore the effects and possible mechanism of ECFCs-exo stimulated by ECSW on H/R injury *in vitro*. With the next-generation sequencing, exosomal miR-140-3p and predicted pathway were selected for further verification. These findings might provide new insights on ECSW therapy for developing exosome derived from ECFCs for myocardial IRI.

## Materials and methods

### Isolation and Culture of Endothelial Colony-Forming Cells

The study was approved by the Animal Care and Use Committee of Kunming Medical University (approval no. kmmu2021109) and performed in accordance with the Guide for the Care and Use of Laboratory Animals. Bone marrow-derived endothelial colony-forming cells were isolated from 4-week-old SD rats using density-gradient centrifugation with Ficoll, and then the cells were inoculated in a culture plate coated with fibronectin. The cells were cultured in induced medium (EGM™-2MV, Lonza, United States) containing VEGF, R3-IGF-1, hEGF, hFGF-B, hydrocortisone, ascorbic acid, GA-1000, and 10% fetal bovine exosome-depleted serum (System Biosciences Inc., United States), placed in a 37°C, 5% CO_2_ incubator. After 72 h, nonadherent cells were removed, and the medium was changed every 3 days. The cell growth characteristics and morphological changes were continuously observed using inverted microscope. The fourth to sixth passage of ECFCs were used for exosomes preparation.

### Identification of Endothelial Colony-Forming Cells

After 14 days of cell culture, the ECFCs were incubated with 20 ug/ml of Dil-acetylated low-density lipoprotein (Dil-Ac-LDL; Maokang Biotechnology, China) for 4 h at 37°C and 5% CO_2_, and then fixed with 4% paraformaldehyde for 20 min. Next, the cells were incubated with 10 µg/ml of FITC-labeled Μlex Europaeus Agglutinin I (FITC-UEA I; Maokang Biotechnology, China) for 1 h at room temperature. Finally, the nuclei were stained with DAPI (4′,6-diamidino-2-phenylindole, Sigma, USA), and the cells were visualized with fluorescence microscope (Olympus IX53, Japan). ECFCs were double stained due to the characteristics of Dil-Ac-LDL uptake and FITC-UEA I binding. Cells were trypsinized, fixed with 4% paraformaldehyde, and blocked using 5% BSA (Solarbio Biotechnology, China) at room temperature for 20 min. Cells were incubated with the primary antibodies against CD31, CD34, CD133, VEGFR-2, and CD14 (Bioss Biotechnology, China), FITC-labeled goat antirabbit lgG antibody, Pacific Blue™ antirat CD45 Antibody (BioLegend, United States), and isotype control antibodies. The cells were washed and resuspended by phosphate-buffered saline (PBS). The percentages of stained surface markers of ECFCs were evaluated by flow cytometry analyses (PARTEC, CyFlow Space, Germany). Tube formation assay of ECFCs was evaluated using Matrigel Basement Membrane Matrix (Corning, USA). Briefly, 50 µl of Matrigel solution was pipetted in a precooled 96-well plate and was incubated at 37°C for 1 h. ECFCs (2 × 10^4^ cells/well) were seeded onto the solidified matrix with 100 µl of culture medium and incubated for 4 h. Tube formations were observed under an inverted fluorescence microscope.

### Extracorporeal Cardiac Shock Wave Processing

The endothelial colony-forming cells were divided into two groups: control group (CON group) and shock wave group (SW group). When the ECFCs growth reached 70%–80% confluency, the SW group was intervened by 500 shots of extracorporeal cardiac shock waves with an energy of 0.09 mJ/mm^2^ (about 10% of the energy used for lithotripsy treatment) using the ECSW system (MODULITH SLC, STORZ MEDICAL, Switzerland) as described in our previous studies (Ma et al., 2020). Simultaneously, the CON group was conducted with experiments without the ECSW treatment. The cells were then cultured for 48 h at 37°C and 5% CO_2_ until the cell culture medium was collected for exosome preparation.

### Exosome Harvesting and Identification

The cell culture medium of ECFCs was collected as described previously, centrifuged at 2,000 ×* g* for 30 min, then the supernatant was filtrated through a sterile 0.22-μm filter to remove cell debris and the large extracellular vesicles. Exosomes were purified from the culture medium using ExoQuick-TC Kit (System Biosciences Inc., USA) according to the instructions of the manufacturer. Briefly, the medium was mixed with 1/5 volume of exosome isolation reagent thoroughly and incubated overnight at 4°C. Exosomes were then harvested by centrifuging the mixture for 30 min at 1,500 × *g* at 4°C. The exosome pellets at the bottom of the tubes were resuspended in 100 μl of PBS solution and stored at −80°C for further study. Exosomes were identified by nanoparticle tracking analysis (NanoFCM, N30E), exosome marker expression, and transmission electron microscopy (Hitachi, HT-7700, Japan). Nanoparticle tracking analysis was used to detect the particle size distribution and concentration information. The exosome markers CD9, CD63, CD81, and calnexin were identified by Western blot assay. The nano-ultrastructure of exosomes were observed by transmission electron microscopy. The protein concentration of exosomes was evaluated by the bicinchoninic acid (BCA) assay kit (Thermo Fisher Scientific, USA).

### Exosome Uptake Assessments

After harvesting the purified exosomes derived from endothelial colony-forming cells, the exosomes were labeled with the PKH26 kit (Sigma-Aldrich, USA) following the instruction manual. H9c2 cells were directly incubated with PKH26 for 5 min as positive control, or for 6 h at 37°C with PKH-26-labeled exosomes and unlabeled exosomes. Then the cells were fixed with 4% paraformaldehyde and stained with DAPI. The red fluorescence signal of PKH26 in the cardiomyocytes was visualized with a fluorescence microscope.

### Hypoxia/Reoxygenation Treatment

The H9c2 cell line, an original clonal cell line derived from embryonic rat heart tissue, was obtained from the BeNa Culture Collection (BNCC, Beijing, China). H9c2 cell line exhibits many of the properties of skeletal muscle and has long been used as a model for cardiac cells in a vast number of studies, particularly the ones intending to address cardiac ischemia/reperfusion injury (Cselenyák et al., 2010; Sousa et al., 2016). The H9c2 cells are routinely cultured in Dulbecco’s Modified Eagle’s Medium (DMEM)/F12 (HyClone, USA) containing 10% fetal bovine exosome-depleted serum at 37°C under an atmosphere of 5% CO_2_ and 95% air. The serum medium was removed from the synchronized cardiomyocytes for 12 h, followed by rinses three times with PBS. To establish the H/R injury model, H9c2 cells were cultured to serum-free, glucose-free DMEM (Sigma-Aldrich, USA) and were exposed to a tri-gas incubator filled with 95% N_2_ and 5% CO_2_ for 4 h at 37°C (hypoxia phase). Then H9c2 cells were returned to the DMEM/F12 containing 10% fetal bovine exosome-depleted serum and incubated under normal conditions of 5% CO_2_ and 95% air at 37°C for 12 h (reoxygenation phase). After H/R injury, H9c2 cells were treated with ECFC-derived exosomes (SW-exo and CON-exo) or PBS for 24 h. H9c2 cells that were cultured in normal DMEM under normoxic conditions were used as control. The divided experimental groups were as follows: NC group (normal control), H/R group, H/R + PBS group, H/R + CON-exo group, and H/R + SW-exo group. The cells were lysed in RIPA lysis buffer containing 1% protease and phosphatase inhibitors (Beyotime Biotechnology, China) and centrifuged; the supernatant and pellets were respectively used for related assays.

### Cell Viability Assay

The cell viability was assessed using the commercially available cell counting kit-8 (CCK-8) (Dojindo Laboratory, Kyushu, Japan). The H9c2 cells were seeded in a 24-well plate (2 × 10^4^ cells/well, 500 µl/well), incubated with exosomes at different concentrations or transfected with miR-140-3p mimic, inhibitor, or negative control (NC), respectively, for 24 h after H/R injury. Then 50 µl of CCK-8 solution was added to each well, and the plates were placed in a 37°C, 5% CO_2_ incubator for 2 h. The supernatant was transferred to a 96-well plate, and the optical density (OD) at 450 nm was measured with a SpectraMax 190 (Molecular Devices, USA).

Lactate dehydrogenase (LDH) activity was measured from the cell culture supernatants as a marker of cell injury by LDH activity kit (Solarbio, China). After the H9c2 cells were grouped and processed (1 × 10^6^ cells/group), the supernatant was collected and centrifuged at 500 × *g* for 5 min to remove cell debris. According to the instructions of the manufacturer, the reagents for detecting LDH activity were added to each sample and mixed thoroughly. The optical density (OD) of the samples at 450 nm was measured by a SpectraMax 190, then the LDH activity was further calculated by a relevant formula.

### Cell Apoptosis Assay

Following treatment, the apoptosis rate of H9c2 cells was analyzed by flow cytometry analyses using the Annexin V-FITC/PI Cell Apoptosis Detection Kit (Dojindo Laboratory, Kyushu, Japan) according to the instructions of the manufacturer. The H9c2 cells were trypsinized and collected together with the floating cells from the medium, washed twice with PBS, resuspended with 1× Annexin V Binding buffer (1 × 10^6^ /ml). Then 5 μl of FITC-labeled Annexin V and 5 µl of propidium iodide (PI) solution were added to 100 µl of cell suspension, mixed gently, and incubated in the dark for 15 min at room temperature. After that, 400 µl of buffer was added to the cell suspension and then analyzed using flow cytometry (PARTEC, CyFlow Space, Germany) within 1 h.

### Cellular Oxidative Stress Detection

The reactive oxygen species (ROS) assay kit (Mao Kang Biotechnology, China) was used to detect the level of ROS *in vitro* by DCFH-DA staining. Briefly, H9c2 cells were seeded in a six-well plate, incubated with 2,7-dichlorodihydrofluorescein diacetate probe (DCFH-DA) (10 μM) at 37°C for 20 min in the dark. After fully reacting with the probe, H9c2 cells were washed three times with serum-free DMEM medium, then observed for intracellular fluorescence of the DCF with a fluorescence microscope (Olympus IX53, Japan) in a blinded manner.

### Next-Generation Sequencing

For SW-exo and CON-exo, next-generation sequencing was performed by LC-Bio Technology Co. Ltd. (Hangzhou, China) in accordance with the standard procedures provided by Illumina, including miRNA sequencing library preparation, sequencing process, and NGS data analysis. Total exosomal RNA was extracted from the exosomes previously purified using Exosomal RNA Isolation Kit (Cat. # 58000, Norgen, Canada) according to the protocol of the manufacturer. The quality and quantity of isolated RNA were evaluated with a Bioanalyzer 2100 instrument (Agilent Technologies, USA). The miRNA sequencing library was prepared using the TruSeq Small RNA Sample Prep Kits (Illumina, United States). The small RNAs ligated with specific RNA adapters to both the 3′ and 5′ ends were performed with reverse transcription, PCR amplification, and gel purification for DNA library construction, followed by sequencing on Illumina Hiseq 2500. Raw reads were subjected to an in-house program, ACGT101-miR, to remove adapter dimers, junk, low complexity, common RNA families (rRNA, tRNA, snRNA, snoRNA), and repeats. Subsequently, unique sequences with lengths in 18–26 nucleotides were mapped to specific species precursors in miRBase 22.0 by BLAST search to identify known miRNAs and novel 3p- and 5p-derived miRNAs.

### Cell Transfection

H9c2 cells were seeded in a six-well plate and cultured in an incubator at 37°C, 5% CO_2_ until 60%–70% confluency. H9c2 cells were respectively transfected with miR-140-3p mimic (sense: 5′-UAC​CAC​AGG​GUA​GAA​CCA​CGG-3′; antisense: 5′-CCG​UGG​UUC​UAC​CCU​GUG​GUA-3′), mimic negative control (NC) (5′-UUU​GUA​CUA​CAC​AAA​AGU​ACU​G-3′, antisense: 5′-CAG​UAC​UUU​UGU​GUA​GUA​CAA​A-3′), miR-140-3p inhibitor (5′-CCG​UGG​UUC​UAC​CCU​GUG​GUA-3′), and inhibitor negative control (NC) (5′-CAG​UAC​UUU​UGU​GUA​GUA​CAA​A-3′) synthesized by Guangzhou RiboBio Company (China). The above oligonucleotides products transfected H9c2 cells for 24 h by using the riboFECT CP Transfection Kit (RiboBio, China) as directed by the protocol of the manufacturer. Briefly, the oligonucleotides were diluted with 1× riboFECT CP buffer, then added with riboFECT CP reagent and mixed gently. The mixture was added to the culture medium (final mimic concentration, 50 nM; final inhibitor concentration, 100 nM). SqRT-PCR was used to verify the transfection effects by detecting the levels of miR-140-3p and target gene. The divided experimental groups were as follows: H/R group, H/R + miR-140-3p mimic NC group, H/R + miR-140-3p mimic group, H/R + miR-140-3p inhibitor NC + SW-exo group, and H/R + miR-140-3p inhibitor + SW-exo group. The cells were lysed and centrifuged, and the supernatant and pellets were respectively used for related assays.

### Luciferase Reporter Assay

According to TargetScan7.2 (https://www.targetscan.org/vert_72/), it is predicted that PTEN 3′UTR may be the potential binding site of miR-140-3p. HEK293 cells were cultured in a 24-well plate and transfection was performed until 70% cell confluency. The HEK293 cells were cotransfected with the constructed PTEN 3′UTR vectors and miR-140-3p mimic or miR-NC using HG Transgene Reagent (Genomeditech Co., China). Briefly, 100 ng of wild-type plasmid PTEN 3′-UTR (WT-PTEN, 5′-GAA​AUU​GUU​CAC​UAG​CUG​UGG​UC) or mutation type plasmid PTEN 3′-UTR (MUT-PTEN, 5′-GAA​AUU​GUU​CAC​UAG​AGU​GUU​GC) was successfully constructed in the vector PGL-CMV-LUC-MCS, and then cotransfected the HEK293 cells with 30 nmol/L miR-140-3p mimic or miR-NC for 48 h. The dual-luciferase reporter gene assay kit (Genomeditech Co., China) was used to detect luciferase activity as directed by the instructions of the manufacturer. With Renilla luciferase as the internal reference gene, the ratio of Luc/Rena was calculated for evaluating the activation of the reporter gene.

### Semi-Quantitative RT-PCR

Total RNA was extracted from cells and exosomes by using TRIzol™ Reagents (Invitrogen, USA). The Mir-X miRNA First-Strand Synthesis Kit (Takara, Japan) was applied for the RNAs reverse transcription, and the Mir-X miRNA qRT-PCR TB Green Kit (Takara, Japan) was used to quantify miRNAs. The extracted RNA was purified and reversely transcribed to cDNA using the TaKaRa PrimeScript™ RT reagent kit with gDNA Eraser (Takara, Japan) as a template in PCR reactions with gene-specific primer pairs (Table 1) using TB Green Premix Ex Taq™ II (Tli RNaseH Plus) (Takara, Japan). SqRT-PCR was performed on QuantStudio 6 Flex Real-Time PCR System (Applied Bio systems, USA) under the following reaction conditions: 95°C for 30 s, 40 cycles at 95°C for 5 s, and 60°C for 34 s. The relative expression levels of miR-140-3p and PTEN expression were determined with the 2^−ΔΔCt^ method. U6 or GAPDH was used as the endogenous controls of miR-140-3p or PTEN, respectively.

**TABLE 1:** The sequences of all primers.

Name of primer	Sequences
miR-140-3p	Forward: 5′-TAC​CAC​AGG​GTA​GAA​CCA​CGG-3′
U6	Forward: 5′-CTC​GCT​TCG​GCA​GCA​CA-3′
	Reverse: 5′-AAC​GCT​TCA​CGA​ATT​TGC​GT-3′
PTEN	Forward: 5′-ATT​CCC​AGT​CAG​AGG​CGC​TA-3′
	Reverse: 5′-TCA​CCT​TTA​GCT​GGC​AGA​CC-3′
GAPDH	Forward: 5′-ACG​GCA​AGT​TCA​ACG​GCA​CAG-3′
	Reverse: 5′-CGA​CAT​ACT​CAG​CAC​CAG​CAT​CAC-3

### Western Blotting

Total proteins were extracted from H9c2 cell lysate or ECFCs-exo using RIPA lysis buffer containing 1% protease and phosphatase inhibitors. The protein concentration was determined by BCA protein assay kit. Protein extracts of each group were run on 10%–12% sodium dodecyl sulfate (SDS) polyacrylamide gels (Beyotime Biotechnology, China) and then transferred to PVDF membranes (Millipore, USA). The membranes were blocked with 5% BSA at room temperature for 2 h and incubated at 4°C overnight with primary antibodies against CD9 (1:1,000, 20597-1-AP, Proteintech), CD63 (1:1,500, AF5117, Affinity), CD81 (1:1,500, ab109201, Abcam), calnexin (1:1,000, AF5362, Affinity), Bax (1:1,000, #2772, Cell Signaling Technology), Bcl-2 (1:1,000, AF6139, Affinity), cleaved caspase-3 (1:1,000, #9664, Cell Signaling Technology), PTEN (1:1,000, #9188, Cell Signaling Technology), PI3K (1:1,000, AF6241, Affinity), phospho-PI3K (1:1,000, AF3242, Affinity), AKT (1:1,000, #4691, Cell Signaling Technology), phospho-AKT (1:1,000, #4060, Cell Signaling Technology), NF-κB (1:1,000, #8242, Cell Signaling Technology), and GAPDH (1:1,000, #5174, Cell Signaling Technology), followed with horseradish peroxidase-conjugated secondary antibodies (1:5,000, Cat.#S0001, Affinity) for 2 h at room temperature. Protein bands were visualized by enhanced chemiluminescence (ECL, Millipore, USA) and analyzed using ImageJ software (https://imagej.nih.gov/ij/). GAPDH antibody was used to control for unequal loading.

### Statistical Analysis

Data are shown as means ± SD of at least three repeated experiments. Shapiro–Wilk test or QQ plot were used to evaluate normality of data. A value of *p *> 0.05 was considered as normal distribution. One-way ANOVA followed by the Tukey’s *post-hoc* test was conducted among multiple groups comparisons, and unpaired Student t-test was carried out to analyze the differences between two groups. A value of *p* < 0.05 was considered a statistically significant difference. All statistical analyses were performed using GraphPad Prism 8.0 (GraphPad Software, Inc., USA).

## Results

### Characterization of Exosomes Derived From Endothelial Colony-Forming Cells

**FIGURE 1:**
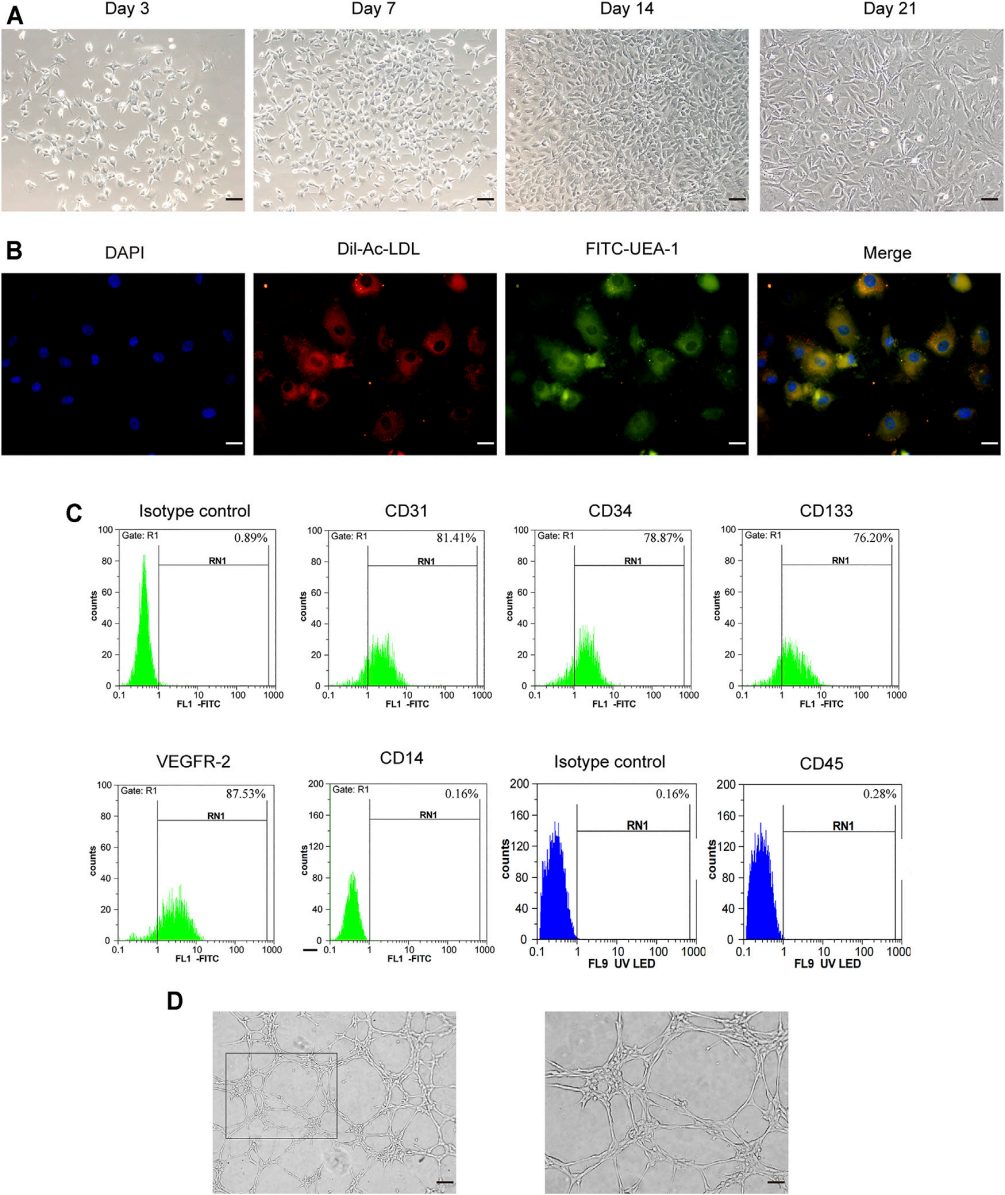
Characterization of rat bone-derived endothelial colony-forming cells (ECFCs). **(A)** On day 3, day 7, day14, and day 21 of cellular morphology under an inverted microscope, scale bar = 100 μm. **(B)** Double staining of ECFCs positive for Dil-AcLDL and FITC-UEA-1 under fluorescent microscope, scale bar = 20 μm. **(C)** Cell surface markers (CD31, CD34, CD133, VEGFR-2, CD14, and CD45) of ECFCs were identified by flow cytometry. **(D)** Tube formation assay of ECFCs was evaluated under an inverted microscope, left: scale bar = 100 μm; right: scale bar = 50 μm.

As shown in Figure 1A, the endothelial colony-forming cells (ECFCs) isolated from rat femoral bone marrow were cultured in EGM™-2MV medium. After removing non-adherent cells, the adherent cells began to adhere to the wall in 3–4 days, showing oval, polygonal, and spindle-shaped changes. On the seventh day, the number of cells obviously increased and showed a colony-like growth. The typical “paving stone"-like cell colonies appeared within 14 days, and the cells exhibited a long fusiform shape on the 21st day. Fluorescence microscopy showed that ECFCs were double-stained attributed to the ability of Dil-Ac-LDL uptake and FITC-UEA I binding (Figure 1B). At the same time, flow cytometry assay showed that the surface markers of endothelial lineage such as CD31, CD34, CD133, and VEGFR2 were all positive (Figure 1C), while hematopoietic markers CD14 and CD45 were negative. Tube formation assay showed that these cells exhibited the formation capillary-like structures with the presence of meshes and branch points, as well as tube-like segments (Figure 1D). The obtained cells were identified by cell morphology, cell surface marker detection, and cell angiogenic function, which were akin to endothelial colony-forming cells.

**FIGURE 2:**
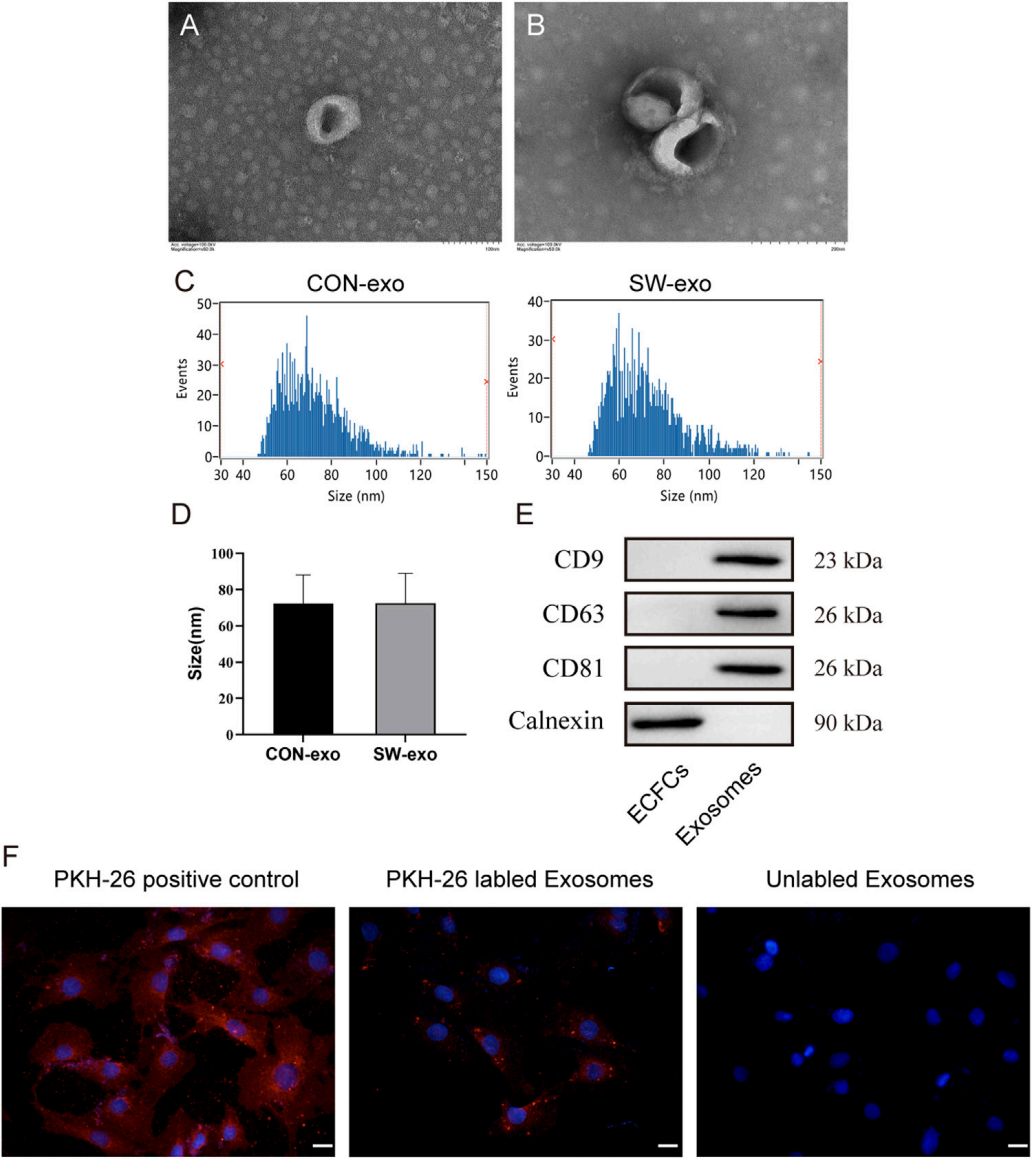
Characterization of exosomes derived from ECFCs. **(A-B)**. exosome morphology was evaluated *via* transmission electron microscopy, scale bar = 100–200 nm. **(C-D)** The size distribution of exosomes was analyzed by nanoparticle tracking analysis. The diameter of exosomes ranges mainly from 30 to 150 nm. **(E)** Western blot analysis of protein levels of CD9, CD63, CD81, and calnexin in cell lysis and ECFCs-exo. **(F)** H9c2 cells were incubated with PKH26 (positive control), PKH26-labeled exosomes, and unlabeled exosomes, nuclei were counterstained with DAPI, and the red fluorescence of PKH-26 in cells was traced by fluorescence microscope, scale bar = 20 μm.

Exosomes were purified from the culture supernatant of ECFCs *via* precipitation in polyethylene glycol. Then the morphology and phenotypes of isolated particles were identified according to the characteristics of exosomes. Transmission electron microscopy showed that the isolated exosomes had double-layer membranes, with complete morphology, uniform size, and spherical or cup-shaped structures (Figures 2A, B). Nanoparticle tracking analysis showed that the size distribution profiles of SW-exo and CON-exo were physically homogeneous with a peak diameter of 72 nm, and the particle concentration is about 7 × 10^8^ particles/ml (Figures 2C, D). In addition, Western blot analysis confirmed that the particles expressed CD9, CD63, and CD81 as widely recognized exosome-specific markers, while the protein calnexin is present in cells and not in exosomes (Figure 2E). Therefore, these results proved that we have successfully extracted exosomes derived from ECFCs. In order to verify the uptake of ECFCs-exo by cardiomyocytes, PKH26(red)-labeled ECFCs-exo were cocultured with H9c2 cells for 6 h. The fluorescence imaging showed that ECFCs-exo could be endocytosed by cardiomyocytes (Figure 2F), which provided the basis for follow-up studies.

### Extracorporeal Cardiac Shock Wave-Induced Endothelial Colony-Forming Cell-Derived Exosomes Protect Cardiomyocytes Against Hypoxia/Reoxygenation Injury

**FIGURE 3:**
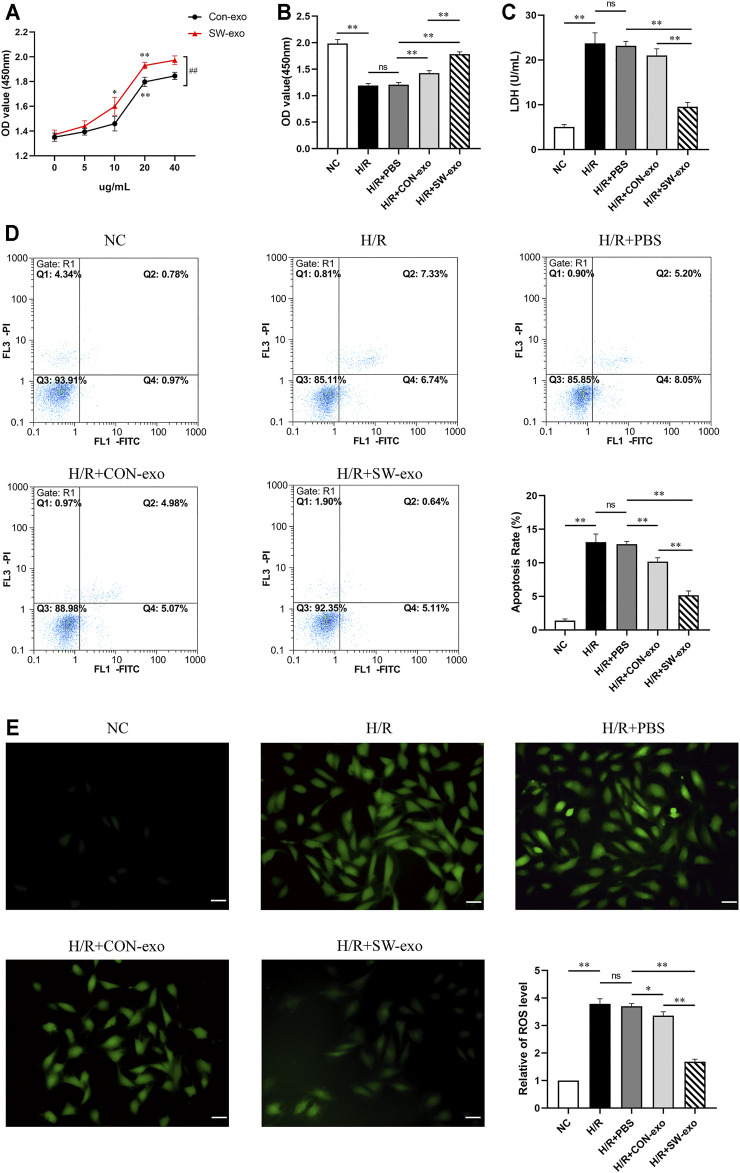
Extracorporeal cardiac shock wave (ECSW)-induced ECFCs-exo protected cardiomyocytes against hypoxia/reoxygenation injury. **(A)** The cell viability of H9c2 cells after hypoxia/reoxygenation (H/R) injury treated with CON-exo and SW-exo at different concentrations from 0 to 40 µg/ml. *p < 0.05, **p < 0.01, within group; ##*p* < 0.01, between groups. **(B–E)** Quantitative analysis of the cell viability **(B)** by cell counting kit-8 (CCK-8) assay, lactate dehydrogenase (LDH) activity **(C)** by reagent kit, cell apoptosis rate **(D)** by flow cytometry, and the production of reactive oxygen species (ROS) **(E)** by dichlorodihydrofluorescein diacetate (DCFH-DA) staining were shown for each group, including NC, H/R, and H9c2 cells after H/R injury treated with PBS, CON-exo, and SW-exo. Data are presented as mean ± SD, n = 3. *p < 0.05, **p < 0.01, E: scale bar = 50 μm.

To explore the effects of ECFCs-exo induced by ECSW, ECFCs-exo treated with shock wave (SW-exo) or without shock wave (CON-exo) were incubated with H9c2 cells after H/R injury. First, we set different concentration gradients of ECFCs-exo from 0 to 40 µg/ml when cocultured with impaired H9c2 cells. As shown in Figure 3A, both SW-exo and CON-exo can improve cell viability, and present in a dose-dependent manner. When the concentration reached to 20 µg/ml, the effects of SW-exo and CON-exo on cell viability were both significantly enhanced (*p* < 0.01), and SW-exo showed more powerful effect than CON-exo (*p* < 0.01). There were no differences in cell viability between 20 and 40 µg/ml; based on that, 20 µg/ml was determined as the optimal exosome concentration and used for further experiments. H/R caused a decrease in cell viability and an increase in LDH activity (*p* < 0.01). Compared with PBS and CON-exo, SW-exo exhibited significantly improved effects on cell viability (*p* < 0.01) (Figures 3B, C). In addition, Annexin-V-FITC/PI double staining by flow cytometry revealed that SW-exo markedly inhibited H9c2 cell apoptosis triggered by H/R (*p* < 0.01), indicating an antiapoptotic effect (Figure 3D). For the detection of ROS in cells, the production of intracellular ROS was significantly increased under H/R condition, while SW-exo treatment suppressed ROS formation (*p* < 0.01) (Figure 3E).

**FIGURE 4:**
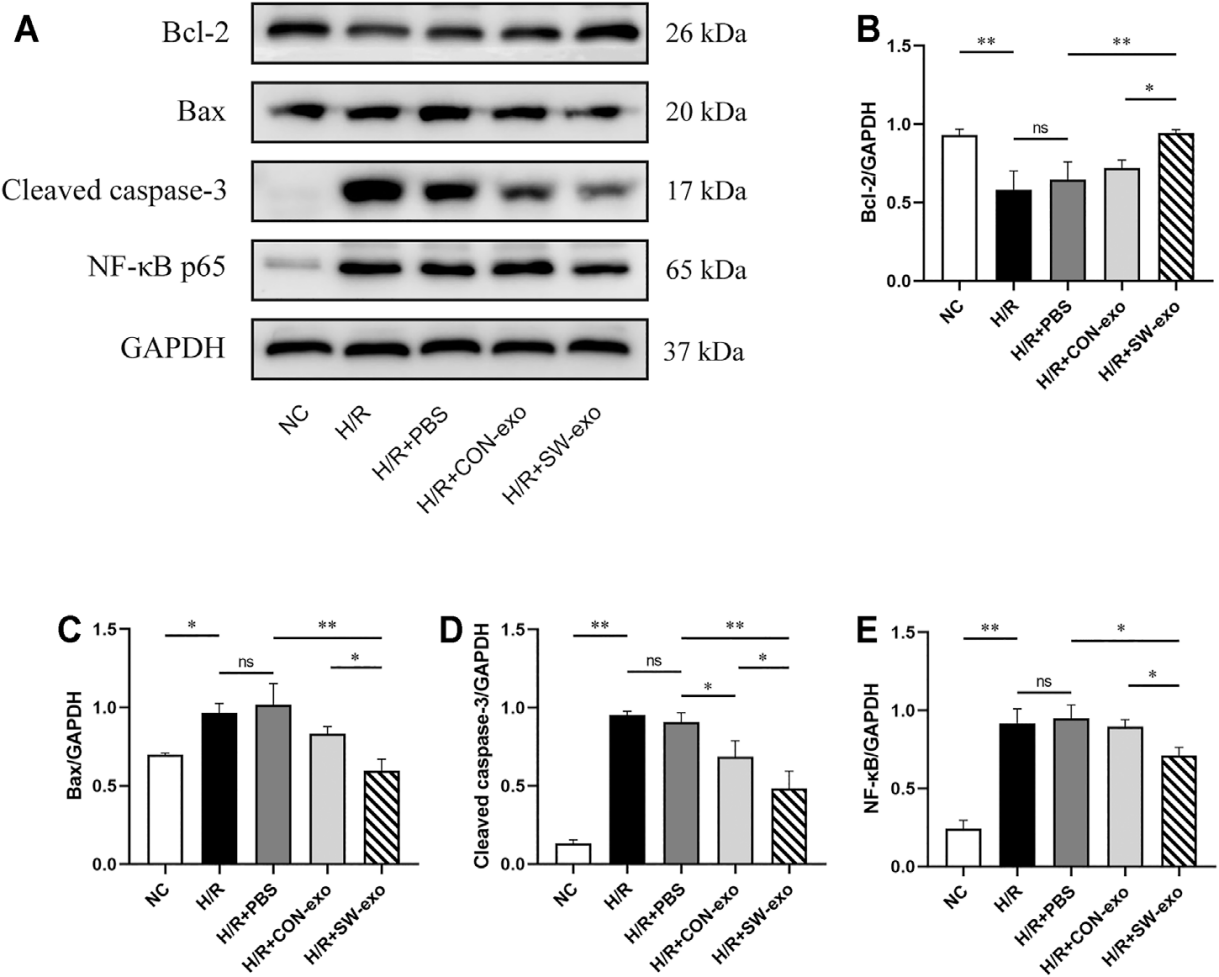
ECSW-induced ECFCs-exo promoted the expression of Bcl-2 and inhibited the expressions of Bax, cleaved caspase-3, and NF-κB p65 in H9c2 cells. **(A)** Western blotting was used to detect the expression of Bcl-2, Bax, cleaved caspase-3, and NF-κB p65 in cardiomyocytes, normalized to the GAPDH protein. **(B–E)** Protein bands were analyzed using ImageJ software. Data are presented as mean ± SD, n = 3. *p < 0.05, **p < 0.01.

To further confirm the function of SW-exo, the related apoptosis and inflammatory proteins in cardiomyocytes were detected by Western blotting. As shown in Figure 4, after H/R, the expression of Bcl-2 was lower, and the expression of Bax and cleaved caspase-3 was higher than that of the NC group (*p* < 0.05). However, the expression of the above proteins was reversely changed when treated with SW-exo. In comparison with PBS and CON-exo, SW-exo significantly promoted the expression of Bcl-2 and reduced the expression of Bax and cleaved caspase-3 (*p* < 0.05). Nuclear transcription factor-κB (NF-κB) proteins are important biomarkers of intracellular inflammatory response in ischemia–reperfusion injury. In our study, SW-exo significantly hindered the upregulation of NF-κB protein, presenting enhanced inhibitor effect than CON-exo (*p* < 0.05). Taken together, these results suggested that SW-exo can protect H9c2 cells from H/R injury by improving cell viability and alleviating cell apoptosis, oxidative stress, and inflammatory response.

### Characterization of miRNA Sequences in Exosomes and Functional Enrichment Analysis

**FIGURE 5:**
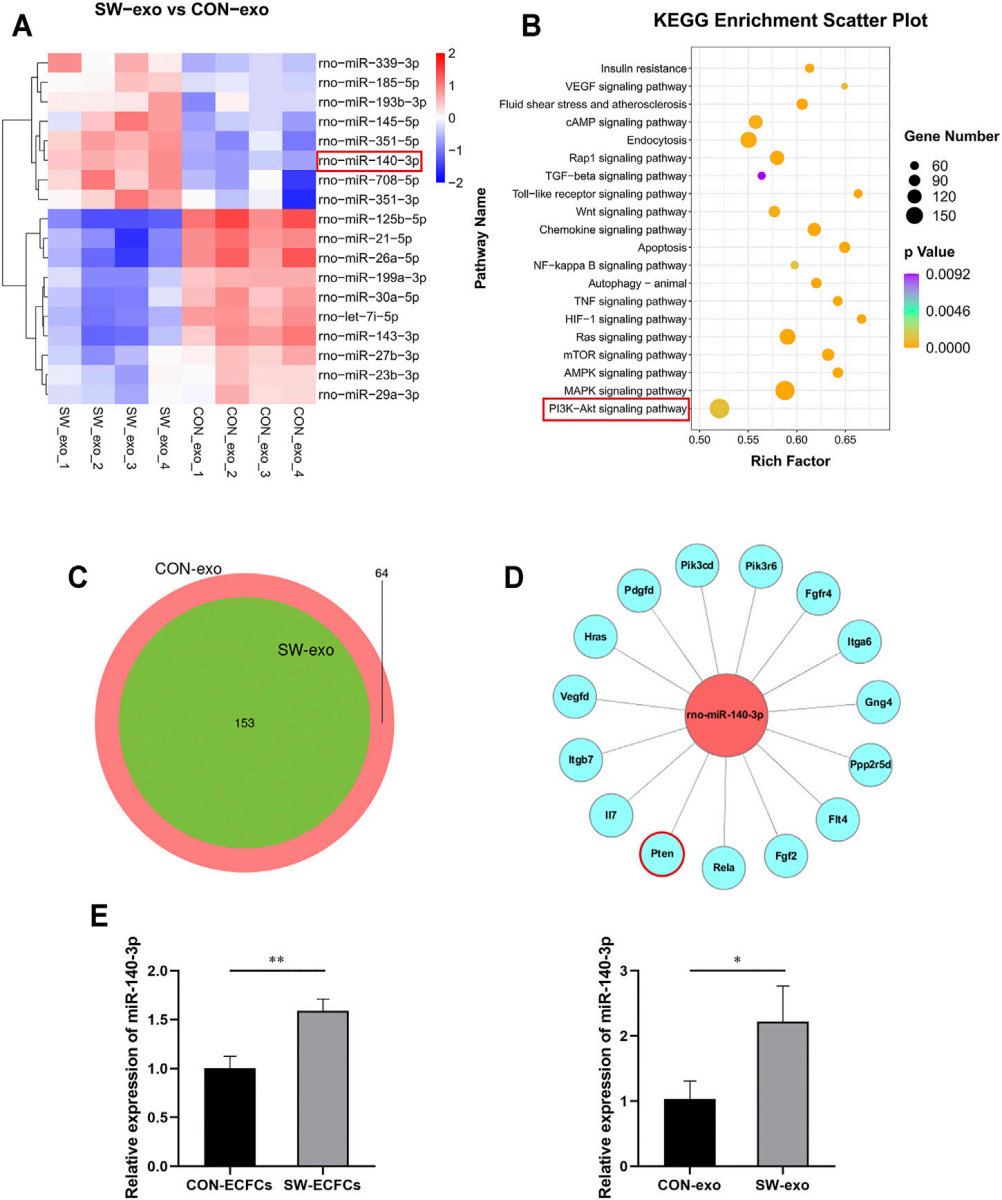
Characterization of miRNA sequences in SW-exo and CON-exo, and functional enrichment analysis. **(A)** Relative amounts of expression for 18 miRNAs enriched in SW-exo and CON-exo are displayed in a heat-map (*p* < 0.05, n = 4). **(B)** Kyoto Encyclopedia of Genes and Genomes (KEGG) functional enrichment analysis of differential target genes was displayed in a scatter plot. **(C)** Venn diagrams of detected miRNAs (SW-exo vs. CON-exo) shows a large overlap between the two groups. **(D)** Mapping of miR-140-3p and target gene network in the PI3K/AKT signaling pathway was drawn by Cytoscape software. **(E)** The relative expression of miR-140-3p in ECFCs and ECFCs-exo were detected by qRT-PCR normalized to U6 (n = 3). *p < 0.05, **p < 0.01.

To further explore the potential molecular mechanism of SW-exo, next-generation sequencing of SW-exo and CON-exo were performed by Illumina Hiseq 2500 to screen the differentially expressed miRNAs and to carry out bioinformatics analysis. In the light of animal species, TargetScan (Nam et al., 2014) and miRanda (Betel et al., 2010) software were used to predict the target genes of differential miRNAs. Then Gene Ontology (GO) and Kyoto Encyclopedia of Genes and Genomes (KEGG) enrichment analysis were performed to determine involved biological functions and signaling pathways. Our sequencing data showed that the miRNAs expression profiles of SW-exo and CON-exo were basically overlapped, indicating a strong homogeneity of ECFCs origin (Figure 5C). The results showed that 18 miRNAs with relative high amounts were differentially expressed in SW-exo and CON-exo, among which 8 miRNAs were upregulated, while 10 miRNAs were downregulated as shown in Figure 5A (*p* < 0.05). GO and KEGG enrichment analysis found that the target genes predicted for the differential miRNAs were primarily focused on cellular function-associated pathways including the PI3K/AKT, MAPK, apoptosis, NF-κB, HIF-1α, Ras, etc (Figure 5B). Based on the KEGG results, we selected PI3K/AKT signaling pathway with significant enrichment of a great quantity of target genes to further study. From the miRNA–mRNA interaction network, we found that a binding site at the 3′UTR of PTEN was predicted on miR-140-3p by TargetScan7.2 (Figure 5D). What is more, our data exhibited that rno-miR-140-3p was most abundant and prominently upregulated after SW stimuli. With the verification by qRT-PCR, it showed that the relative expression of miR-140-3p was significantly higher both in SW-ECFCs and SW-exo than that in the control group without ECSW (*p* < 0.05) (Figure 5E), which was consistent with the NGS results. These data suggested that ECSW stimulated an overexpression of miR-140-3p in ECFCs-exo possibly *via* inhibiting target gene PTEN to activate downstream PI3K/AKT signals in cell-repairing processes.

### Pivotal Effect of Exosomal miR-140-3p on Repair of Cardiomyocyte Hypoxia/Reoxygenation Injury

**FIGURE 6:**
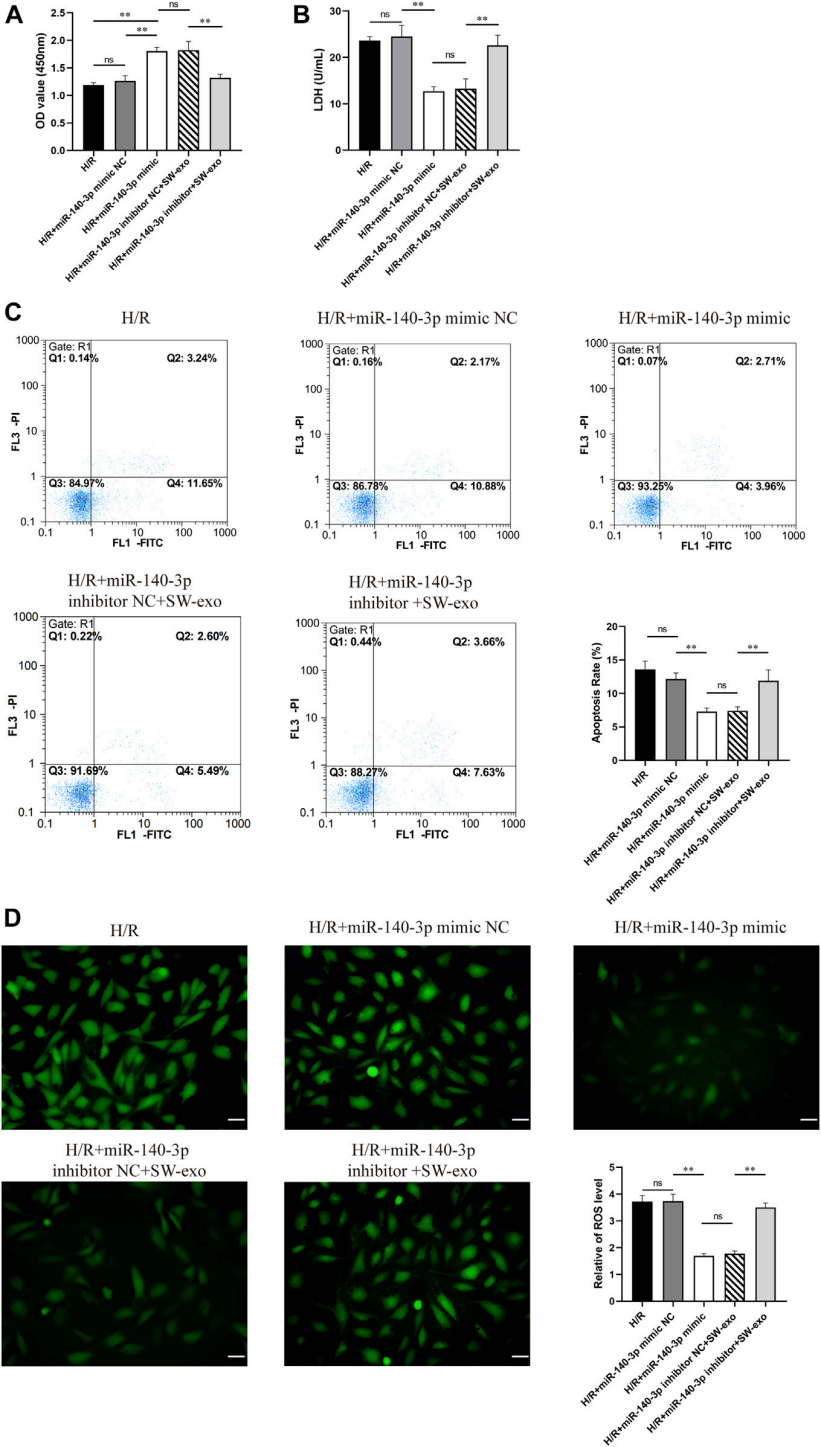
SW-exo mainly delivered pivotal miR-140-3p to protect cardiomyocytes against hypoxia/reoxygenation injury. **(A–D)** Quantitative analysis of the cell viability **(A)** by CCK-8 assay, LDH activity **(B)** by reagent kit, cell apoptosis rate **(C)** by flow cytometry, and the production of ROS **(D)** by DCFH-DA staining are shown for each group, including H9c2 cells after H/R injury treated with miR-140-3p mimic, mimic NC, miR-140-3p inhibitor, inhibitor NC, and SW-exo. Data are presented as mean ± SD, n = 3. *p < 0.05, **p < 0.01, E: scale bar = 50 μm.

**FIGURE 7:**
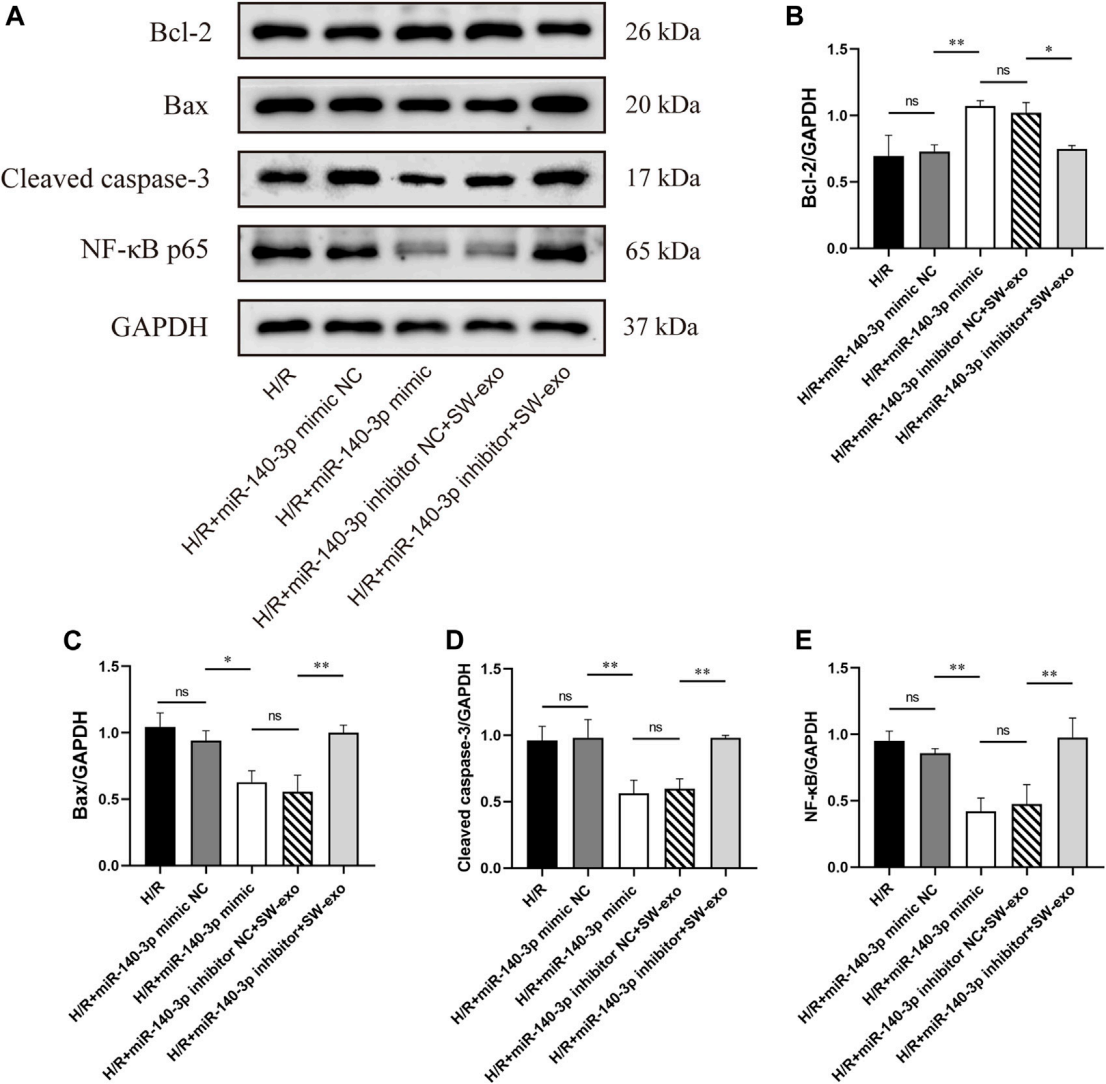
SW-exo delivered pivotal miR-140-3p to H9c2 cells for promoting the expression of Bcl-2 and inhibiting the expressions of Bax, cleaved caspase-3, and NF-κB p65. **(A)** Western blotting was used to detect the expression of Bcl-2, Bax, cleaved caspase-3, and NF-κB p65 in cardiomyocytes, normalized to the GAPDH protein. **(B–E)** Protein bands were analyzed using ImageJ software. Data are presented as mean ± SD, n = 3. *p < 0.05, **p < 0.01.

To confirm the role of miR-140-3p in SW-exo, we impeded the function of miR-140-3p by transfecting H9c2 cells with miR-140-3p inhibitor. Meanwhile, H9c2 cells after H/R were treated with miR-140-3p mimic as positive control. The results demonstrated that miR-140-3p mimic significantly improved cell viability and reduced LDH activity compared with mimic NC (*p* < 0.01), consistent with SW-exo + inhibitor NC. However, the therapeutic effects of SW-exo were mainly blocked by the miR-140-3p inhibitor with a decrease in cell viability and an increase in LDH activity (*p* < 0.01) (Figures 6A, B). In cell apoptosis assay, miR-140-3p mimic significantly attenuated apoptosis of H9c2 cells after H/R compared with mimic NC, while miR-140-3p inhibitor blunted the role of SW-exo and resulted in a higher apoptosis rate (*p* < 0.01) (Figure 6C). The similar change happened in ROS production in H9c2 cells. Results showed that miR-140-3p overexpression restrained the ROS formation, which could be further suppressed by miR-140-3p inhibitor (*p* < 0.01) (Figure 6D). As shown in Figure 7, Western blotting revealed that the expression of antiapoptotic factor Bcl-2 was higher and the proapoptotic factors Bax and cleaved caspase-3 were lower in the miR-140-3p mimic group than that in the mimic NC group (*p* < 0.05). The above apoptosis-related protein exhibited the opposite trend when miR-140-3p inhibitor acted on SW-exo in H9c2 cells (*p* < 0.05). The expression of NF-κB protein was significantly reduced in the miR-140-3p mimic and SW-exo group, which was reversed by miR-140-3p inhibitor (*p* < 0.01). These results further confirmed that ECSW-induced ECFCs-exo mainly delivered miR-140-3p to protect H9c2 cells against H/R injury.

### SW-Exo Containing miR-140-3p Downregulate PTEN Expression

**FIGURE 8:**
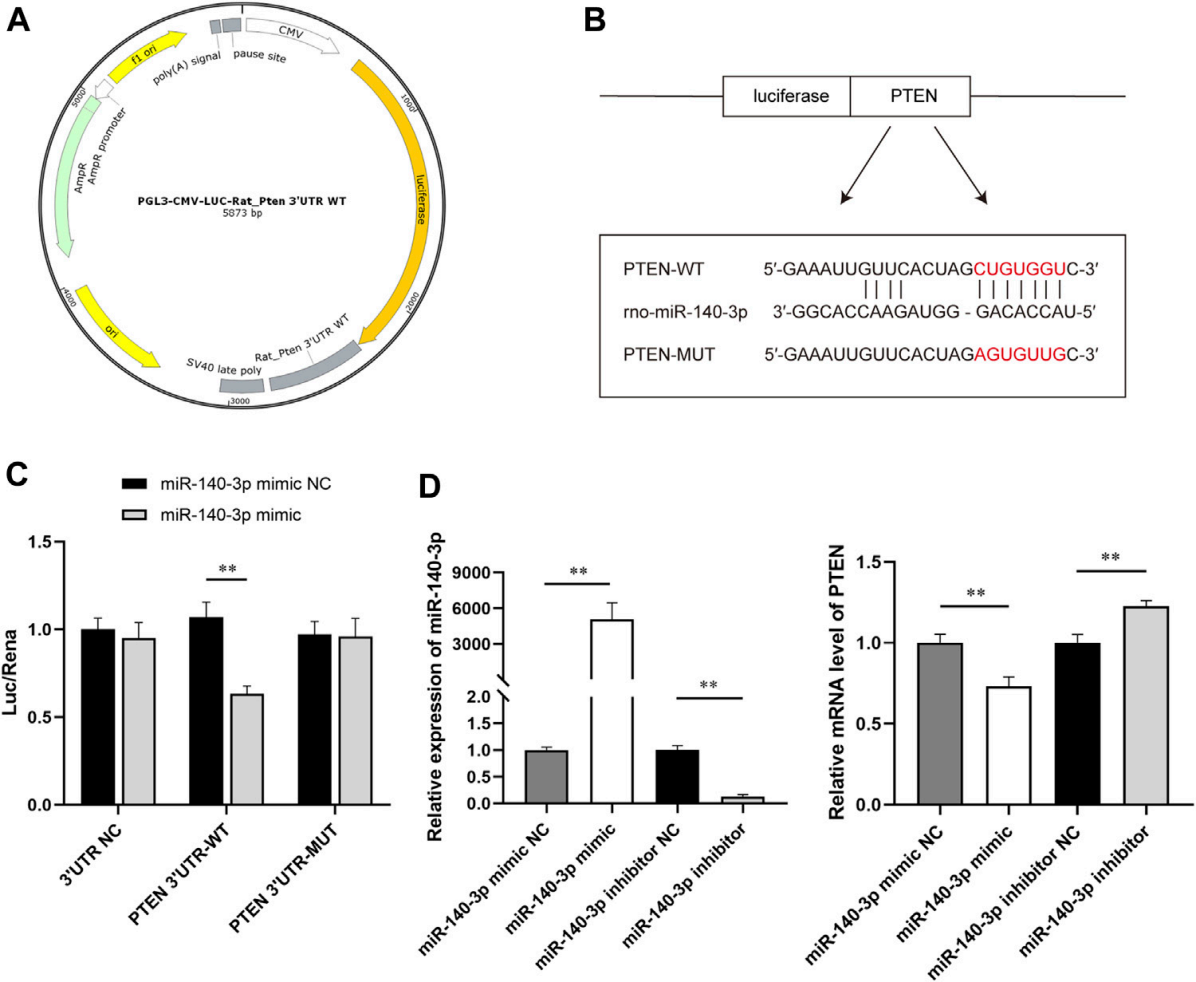
SW-exo containing miR-140-3p inhibited the expression of PTEN. **(A)** Profile of reporter vectors expressing luciferase fused with the 3′UTR of the PTEN wild type (WT) or mutant (MUT). **(B)** Potential binding sites (red) for miR-140-3p on PTEN 3′UTR are predicted, and mutated sequences are presented. **(C)** The relative luciferase activity was measured by dual-luciferase reporter gene assay (n = 3). **(D)** The relative expression of miR-140-3p and PTEN mRNA in H9c2 cells after transfected with miR-140-3p mimic or mimic NC, miR-140-3p inhibitor, or inhibitor NC were detected by qRT-PCR normalized to U6 and GAPDH (n = 3). *p < 0.05, **p < 0.01.

Based on the results of bioinformatics analysis, 3′UTR of PTEN was predicted to be the target site for miR-140-3p. To further investigate the interaction between miR-140-3p and PTEN, we constructed reporter vectors expressing luciferase fused with the 3′UTR of PTEN wild type (WT) or mutant (MUT) (Figure 8A). HEK293 cells were cotransfected with the constructed reporter vectors and miR-140-3p mimic or miR-NC. There were two putative complementary sequences for miR-140-3p in the 3′UTR of PTEN (Figure 8B). The site-directed mutagenesis was used to further determine the exact binding site. The empty vector as a negative control eliminated the interference of the plasmid itself to make the experimental results more reliable. Dual-luciferase reporter assay showed that luciferase activity significantly declined when PTEN 3′UTR-WT combined with miR-140-3p mimic, but not with mimic NC (*p* < 0.01). The activity of the luciferase was not influenced when cotransfected with PTEN 3′UTR-MUT and miR-140-3p mimic (Figure 8C). In addition, H9c2 cells were transfected with miR-140-3p mimic or mimic NC, miR-140-3p inhibitor or inhibitor NC, respectively, for 24 h. qRT-PCR showed that the relative expression of miR-140-3p was extremely higher in the mimic group than in the mimic NC, following a downregulation of PTEN mRNA (*p* < 0.01), which was consistent with the results of the dual-luciferase assay. On the contrary, the transfection of miR-140-3p inhibitor significantly downregulated the level of miR-140-3p and upregulated the level of PTEN mRNA (*p* < 0.01) (Figure 8D). Together, these data suggested that miR-140-3p directly targeted PTEN and inhibited its expression, which subsequently regulated downstream signals to restore the cell function.

### SW-Exo Improves Hypoxia/Reoxygenation Injury *via* the miR-140-3p/PTEN/PI3K/AKT Pathway

**FIGURE 9:**
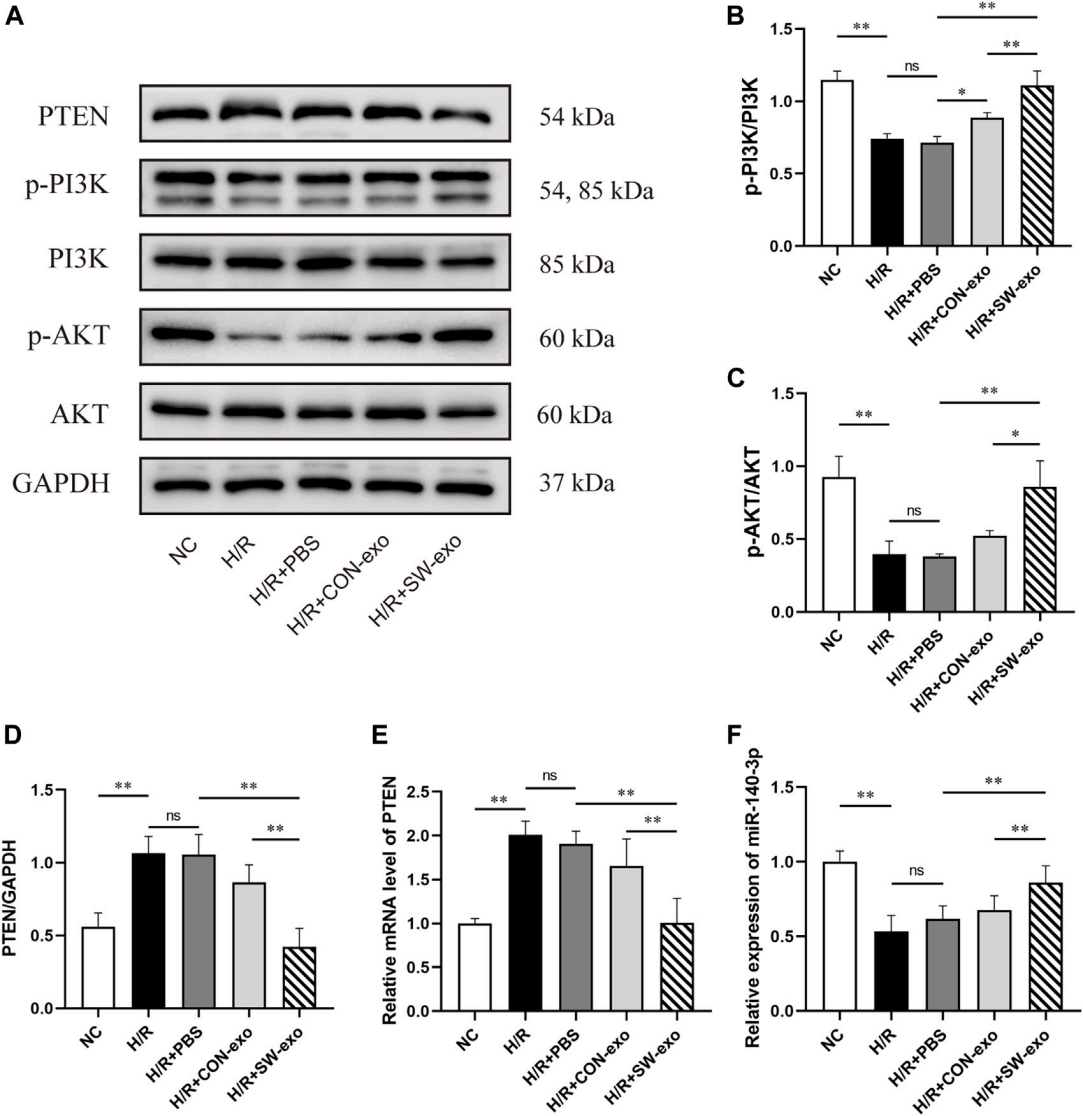
SW-exo exerted regulatory effect on the PTEN/PI3K/AKT signaling pathway *via* miR-140-3p in H9c2 cells. **(A–D)** Western blot was used to detect the expression of PTEN, p-PI3K, PI3K, p-AKT, and AKT, normalized to the GAPDH protein. **(E, F)** The relative expression of miR-140-3p and PTEN mRNA in H9c2 cells were detected by qRT-PCR, normalized to U6 and GAPDH. Data are presented as mean ± SD, n = 3. *p < 0.05, **p < 0.01.

Given that PTEN/PI3K/AKT constitutes an important pathway involved in cellular survival and functions, upregulation of miR-140-3p in SW-exo is possibly related to this signaling pathway. We also assessed the expression of PTEN/PI3K/AKT protein in H9c2 cells after treatment. As shown in Figures 9A–D, the level of PTEN protein was increased after H/R (*p* < 0.01), following a decrease in p-PI3K and p-AKT proteins. Compared with PBS and CON-exo, SW-exo had stronger effects on reducing PTEN protein and inducing p-PI3K and p-AKT proteins, while the expression of total PI3K and AKT were not affected. The ratio of p-PI3K/PI3K and p-AKT/AKT was upregulated by SW-exo (*p* < 0.05), which consequently enhanced the phosphorylation of the PI3K/AKT signaling pathway. qRT-PCR analysis showed that the change in PTEN mRNA was consistent with its protein level, and miR-140-3p was contrary to the trend of PTEN mRNA. In detail, miR-140-3p was low expressed in H9c2 cells after H/R injury, but SW-exo significantly elevated the level of miR-140-3p in the cells (Figures 9E, F).

**FIGURE 10:**
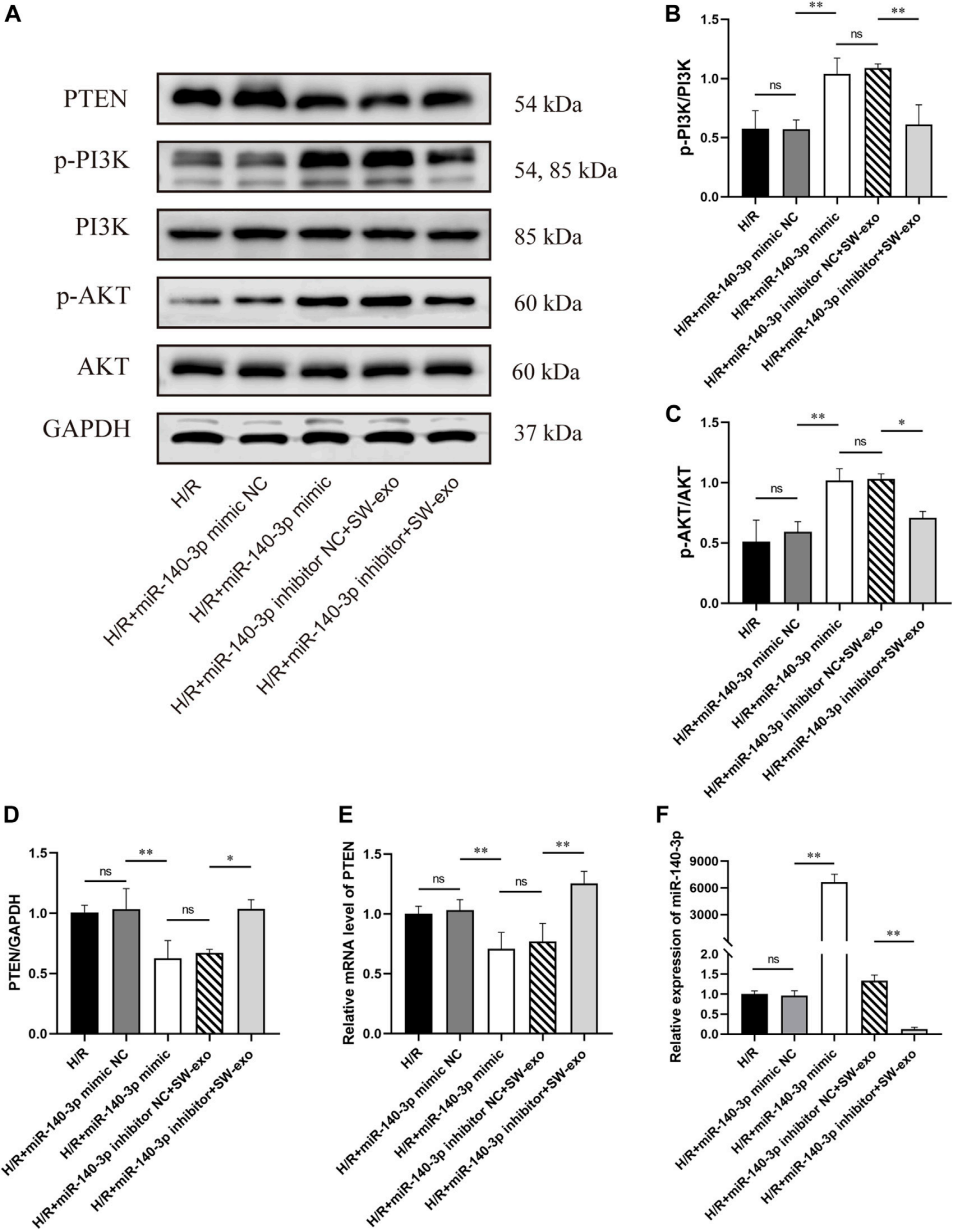
miR-140-3p inhibitor reversed the effect of SW-exo by blunting the PTEN/PI3K/AKT signaling pathway in H9c2 cells. **(A–D)** Western blot was used to detect the expression of PTEN, p-PI3K, PI3K, p-AKT, and AKT, normalized to the GAPDH protein. **(E, F)** The relative expression of miR-140-3p and PTEN mRNA in cardiomyocytes were detected by qRT-PCR, normalized to U6 and GAPDH. Data are presented as mean ± SD, n = 3. *p < 0.05, **p < 0.01.

Besides, we found that the protein level of PTEN was significantly inhibited by miR-140-3p mimic or SW-exo with inhibitor NC, exactly opposite to the trend of p-PI3K and p-AKT (*p* < 0.01). The effects of SW-exo on the PTEN/PI3K/AKT signaling pathway were obviously negated by miR-140-3p inhibitor, indicating higher PTEN proteins and lower ratio of p-PI3K/PI3K and p-AKT/AKT (Figures 10A–D). Subsequently, qRT-PCR assay also showed that the level of miR-140-3p was dramatically elevated, and the expression of PTEN mRNA was declined when treated with miR-140-3p mimic, but not mimic NC (*p* < 0.01). miR-140-3p inhibitor reduced the level of miR-140-3p with an upregulation of PTEN mRNA (*p* < 0.01) (Figures 10E, F). These results indicated that exosomal miR-140-3p mitigated cardiomyocyte H/R injury mainly by mediating a posttranscriptional regulation on the PTEN/PI3K/AKT signaling pathway.

## Discussion

In this study, we first demonstrated that exosomes derived from ECFCs induced by ECSW (SW-exo) appear to enhance cardioprotective effects on H/R injury *in vitro*. We found that SW-exo stood out well against cell apoptosis, oxidative stress, and inflammation triggered by H/R injury. miR-140-3p was identified as the responsible exosome cargo in this process by regulating the PTEN/PI3K/AKT signaling pathway. Our results suggest that ECSW stimuli may confer ECFCs-exo functional cargo *via* delivering to H9c2 cells, thereby showing a curative potentiality.

Following myocardial ischemia/reperfusion, an inflammatory cascade response is triggered by increasing damage-associated molecules including ROS, pro-apoptotic, and pro-inflammatory factors (Bugger and Pfeil, 2020). With the peroxidation of cytomembrane, intracellular Ca^2+^ overload, and mitochondrial dysfunction, reperfusion phase gives rise to further damage on the surviving cardiomyocyte, and even leading to cell death and progressive fibrosis (Neri et al., 2017). A clinical study found that timely starting of low-energy ECSW therapy within 48–72 h in patients with AMI who had successfully undergone PCI exhibited amelioration of left ventricular remodeling and cardiac function, but the specific mechanism is not yet completely elucidated (Kagaya et al., 2017). ECSW has been proven to bring cardiac benefits mostly by mobilizing EPCs in human or animal studies, resulting in recruitment and homing of endogenous EPCs to the damaged ischemic myocardium (Wojakowski et al., 2012). Emerging evidences indicated that the function of EPCs is mainly achieved by secreting bioactive paracrine vesicles in the form of exosomes, promoting cutaneous wound healing (Xu et al., 2020), recovery of brain injury (Wang et al., 2020a), and endothelial dysfunction (Bai et al., 2020). ECFCs are a group of circulating EPCs capable of migratory, self-renewal, and angiogenesis (Yu et al., 2018). ECSW across the body surface could generate a cavitation effect (localized stress on myocardial tissue and cell membranes) (Nishida et al., 2004), which may give a stimulus on homing ECFCs and impact on their exosome secretion.

On the bases of previous findings, we innovatively investigated the effects of ECFCs-exo under ECSW stimulation on cardiomyocytes H/R injury and further explored the underlying mechanism. First, we confirmed that ECFCs-exo can be taken up by H9c2 cells and provided a prerequisite for exosomes therapy. Our previous study found that hypoxia can induce damage in H9c2 cells, which can be further aggravated by reoxygenation, most notable after 12 h (Hu Z. et al., 2018). Exosomes as therapeutic intervention was performed after reoxygenation. Although the two groups of exosomes are similar in particle concentration, microstructure, and phenotypic characteristics, the SW-exo exerted profound therapeutic effects against H/R injury evidenced by improved cell survival, reduced cell apoptosis, ROS production, and inflammatory response. We observed that CON-exo have limited beneficial effects on impaired cardiomyocyte, in accord with previous studies (Yue et al., 2020; Huang et al., 2021). Up to now, ECSW was once reported only in human umbilical vein endothelial cells (HUVEC) to promote exosome cells (Gollmann-Tepeköylü et al., 2020), but not in ECFCs yet. Our finding was different from this earlier study, but the number of exosomes was virtually unchanged post ECSW, probably a change in contents of exosomes. It is well established that external stimuli may alter stem cell–exosome release, content, or function. The contribution of ECFCs-exo in angiogenesis was noteworthy and widely studied in the past several decades, but ECFCs-exo also showed powerful effects on antiapoptosis, antioxidation, anti-inflammation, inhibition of endothelial-to-mesenchymal transition (EndMT), and cardiac fibrosis (Zeng et al., 2021). This study focused on the role of SW-exo in cardiomyocytes H/R injury. Surprisingly, we found that ECSW have significantly amplified the advantages of ECFCs-exo, which may partially explain the possible mechanism of ECSW therapy in CVDs.

Exosomes deliver a wide range of functional cargoes that play critical roles in intercellular information transmission by affecting biological processes of cardioprotection (Zheng et al., 2020). It was reported that a variety of miRNAs were abundant in ECFCs-exo, particularly mediating angiogenesis and repair of myocardial damage (Hu et al., 2019; Zhou et al., 2019; Ke et al., 2021). It has been well acknowledged that miRNAs are highly conservative among human and different animal species such as rats and mice. Here, we performed next-generation sequencing and qRT-PCR verification for CON-exo and SW-exo to screen out the differential miRNAs, among which miR-140-3p was found to be highly plentiful in ECFCs-exo. The exosomal miR-140-3p was not only significantly upregulated after ECSW stimuli but also presented prominently restorative effects on H9c2 cells when suffering from H/R injury. Previous studies have revealed that miR-140-3p was possibly associated with osteoarthritis, liver fibrosis, tumorigenesis, and progression (Ntoumou et al., 2017; Huang et al., 2019; Wu and Li, 2019). However, there is still controversy surrounding the cardioprotective role of miR-140-3p. It was reported that circulating miR-140-3p could be valuable biomarkers for risk estimation in coronary artery disease (Karakas et al., 2017). Elevated plasma miR-140-3p was present during early period of acute coronary syndrome (Li et al., 2017). Meanwhile, another evidence suggested that lower levels of miR-140-3p in patients with CVDs was negatively correlated with risk of cardiovascular events and mortality (Lin and Ma, 2019). Recently, studies of some noncoding RNA in I/R injury indirectly revealed that miR-140-3p may play a functional role in anti-apoptosis, anti-inflammatory, and antioxidative stress (W.W. Wang et al., 2017; Liang et al., 2020; Yi et al., 2020). Similarly, our findings further support miR-140-3p as cardioprotective signals in SW-exo, providing more evidence of future therapeutic strategy for CVDs.

Target gene prediction and dual-luciferase reporter assay revealed that miR-140-3p can directly bind to the site of PTEN mRNA 3′UTR to exert a negative regulatory effect. PTEN has been extensively studied in tumor development and angiogenesis, but increasing evidence demonstrated that PTEN was also closely related to cardiovascular disease (Parajuli et al., 2012). It was reported that the inhibition of PTEN cannot only reduce cardiomyocyte apoptosis, oxidative stress, and DNA damage in ischemic injury (Gong et al., 2021) but also shrink infarction areas and improve cardiac function in MI mice (Feng et al., 2020). We observed that the expression of PTEN in both protein and mRNA in H9c2 cells have risen sharply after H/R injury, but was restrained by SW-exo and miR-140-3p. A recent study showed that patients with AMI were accompanied with an increased level of PTEN in serum (Ling et al., 2020), which was consistent with our finding. In addition, the present data showed SW-exo and miR-140-3p significantly promoted the phosphorylation of the PI3K/AKT pathway in cardiomyocytes, while miR-140-3p inhibitor impeded this activation. To the best of our knowledge, PI3K/AKT is an important signal transduction pathway involving antiapoptosis, pro-angiogenesis, and antiventricular remodeling effects induced by ECSW (Wang et al., 2021). This further supports the cardioprotective effects of SW-exo *via* the miR-140-3p/PTEN/PI3K/AKT pathway in cardiomyocytes. Some evidence indicated that miR-140-3p could indirectly inhibit inflammatory factor p38 MAPK and NF-κB by binding to the 3′UTR of CD38 mRNA (Jude et al., 2012). miR-140 mimic has remarkably lowered the protein expression of NF-κB in myocardial IRI rats (Hao et al., 2020), which was similar to our results. miR-140-3p was evidenced to transcriptionally regulate the hypoxia-inducible factor-1α (HIF-1α), and play antioxidative and cytoprotective roles under ischemic strokes (Yi et al., 2020). Furthermore, the advanced mechanisms by which exosomal miR-140-3p regulates various cellular processes remain to be elucidated.

It has been found that ECSW therapy can bring about high expression of vascular endothelial growth factors (VEGF) and its receptors that rapidly activate AKT and ERK signals to promote angiogenesis in the border zone of the infarcted myocardium (Gollmann-Tepekoylu et al., 2018). Sequencing analysis showed that many of the angiogenic miRNAs were also of significant difference in SW-exo, including proangiogenetic factors (miR-21, miR-30, miR-145, and miR-185) or antiangiogenetic factors (miR-26, miR-27, miR-125, and miR-199), but some are still controversial. The up- or downregulation of these strongly abundant miRNAs in the SW-exo may account for the angiogenic process through a series of complex regulation. It was reported that exosomes from human umbilical cord blood could accelerate cutaneous wound healing through miR-21-3p-mediated promotion of angiogenesis and fibroblast function (Hu Y. et al., 2018). While decreasing miR-199a-3p may alleviate the inhibition for target gene VEGF in favor of angiogenesis in endothelial repair (Ghosh et al., 2017). Indeed, when intervened with exosomes, the recipient cells are affected by a myriad of factors acting simultaneously and finally appear to have a global comprehensive effect. This might be another scope worth further study.

There are several limitations that should be considered in the present study. First, we preliminarily verified the cardioprotective role of SW-exo and miR-140-3p in H/R injury. The contribution of SW-exo and other miRNAs especially angiogenesis was of great potentiality, but it is still with inadequate understanding that deserves to be deeply explored in the future. Second, in this study, we used rat cardiac myoblast cell line *in vitro* model for IR damage and only performed cell experiments with a small sample size. Human iPSC-derived cardiomyocytes and human ECFCs from umbilical cord or peripheral blood are more optimal for further verification. Additionally, an animal model for myocardial IRI is needed to assess therapeutic efficacy of SW-exo in the next work. Third, due to exosomes derived from multifarious cells, it is nearly impossible to isolate circulating ECFCs-exo after SW therapy *in vivo* for a lack of specific surface marker. Fourth, nanoparticle tracking analysis (NTA) showed high variability and measuring error. We, therefore, used BCA assay to evaluate exosome concentration. This study has provided some evidence *in vitro* for proving the effects of SW-exo on H/R injury, but the mechanism by which ECSW-induced ECFCs-exo rescue damaged myocardium *in vivo* remains to be elucidated.

## Conclusion

In conclusion, the current work suggested ECSW as an effective stimulus to activate the function of exosomes derived from ECFCs. SW-exo play a therapeutic role in cardiomyocyte H/R injury with antiapoptosis, antioxidative stress, and anti-inflammatory effects. Exosomal miR-140-3p as a key cardioprotective mediator mainly regulates the PTEN/PI3K/AKT pathway, following suppression of cell damage *via* working with a series of related proteins. These findings present a novel mechanism underlying ECSW-induced ECFCs-exo in cardioprotection and provide an alternative cell-free approach for myocardial IRI.

## Data Availability

The datasets presented in this study can be found in online repositories. The names of the repository/repositories and accession number(s) can be found below: NCBI SRA BioProject, accession no: PRJNA756360.
